# Forces and moments generated during extrusion of a maxillary central incisor with clear aligners: an in vitro study

**DOI:** 10.1186/s12903-023-03136-2

**Published:** 2023-07-17

**Authors:** Abraham McKay, Julie McCray, Brent Bankhead, Michael M. Lee, Gabriel Miranda, Samar M. Adel, Ki Beom Kim

**Affiliations:** 1 Department of Orthodontics, Saint Louis University, Saint Louis, MO USA; 2 Department of Orthodontics, Faculty of Dentistry, Alexandria University, Alexandria, Egypt

**Keywords:** Clear aligners, 3D-printed aligners, Direct printed aligners, Extrusion, Attachments, Pressure columns, Forces and moments

## Abstract

**Objective:**

To assess the possibility of extrusion of a maxillary central incisor with the use of buccal and lingual pressure columns in the absence of attachments, and to evaluate the forces and moments experienced by the teeth using both thermoformed and 3D-printed clear aligners.

**Materials and methods:**

A three-axis force and moment sensor (Aidin Robotics, Anyang, South Korea) was used to measure the forces and moments during extrusion of an upper left central incisor (UL1) and any forces experienced by the upper right central incisor (UR1) using thermoformed aligners and 3D-printed aligners. For the thermoformed aligners, the materials used were ATMOS® (American Orthodontics, Sheboygan, WI) and Zendura FLX® (Bay Materials LLC, Fremont, CA). 3D-printed aligners were fabricated using TC-85 clear photocurable resin (Graphy Inc., Seoul, South Korea). For each material type, three conditions were tested: Group 1: No attachment or pressure columns (control); Group 2: Attachment only; and Group 3: Pressure columns only. Each group was planned for 0.5 mm of extrusion on the UL1.

**Results:**

All force readings collected demonstrated statistically significant differences when compared by materials and when compared by groups, with a *P* value of < 0.001. In the absence of attachment or pressure columns (Group 1), ATMOS® and TC-85 groups exerted extrusive force on the UL1. However, significantly lower forces and moments were exerted by the TC-85 group in comparison to the ATMOS® and Zendura FLX® groups. In the presence of attachment (Group 2), all three ATMOS®, Zendura FLX® and TC-85 groups exerted extrusive force on the UL1, with the TA group showing different directions of faciolingual force, mesiodistal force and faciolingual inclination on the UR1 when compared to the other two thermoformed groups. Whereas in the presence of pressure columns (Group 3), only the TC-85 3D-printed aligner group exerted extrusive force. Thermoformed aligners generated significantly higher mean forces and moments than 3D-printed aligners. Significant levels of unintended forces and moments were present in all groups.

**Conclusions:**

Force levels generated during extrusion with clear aligners are significantly lower with those 3D-printed using TC-85 than with those thermoformed using ATMOS® or Zendura FLX®**.** Attachments consistently generated extrusive forces, and may be an effective adjunct in achieving extrusion of incisors. Extrusion may be achieved without the use of attachments by utilizing pressure columns in 3D-printed aligners using TC-85. While different strategies can generate extrusive forces, there are significant unintended forces and moments.

## Introduction

Since the introduction of Kesling’s tooth positioner in 1944, there has been a significant amount of research and development to improve this initial concept. Orthodontists have built upon the idea of a tooth positioner by using a series of clear retainers, each producing incremental tooth movement, to correct minor misalignment of teeth [1, 2]. In the late 1990s, Align Technology Inc. became the first to successfully market a CAD/CAM-based clear aligner product with the Invisalign® system and the ClinCheck® digital treatment planning platform [3]. Thereafter, a combination of effective marketing, patient demand for invisible orthodontics, and advances in digital technology has led to a widespread adoption of clear aligner therapy as a comprehensive alternative to fixed appliance therapy. [4] Recently, digital technology has become even more accessible and affordable, allowing the orthodontist to digitally treatment plan and fabricate clear aligners within a private practice setting [5].

Due to the increasing popularity of clear aligner therapy, it is imperative to truly understand their efficacy in achieving tooth movement. Initial studies reported the overall accuracy of tooth movement with Invisalign® as 41%, with extrusion being less accurate, at 29.6%. The extrusion of the maxillary central incisor in particular was even less predictable, at 18.3% [6, 7]. Indeed, a systematic review in 2015 agreed that extrusion was among the least predictable movements, at 30% accuracy [8]. To overcome the inherent lack of clinical predictability of clear aligners, different materials, design features and adjunctive strategies have been proposed [9, 10]. By 2020, the overall accuracy of tooth movement with Invisalign® had improved to 50%, whereas the accuracy of extrusion of the maxillary central incisor improved to 56% [7].

Traditionally, clear aligners have been fabricated by the thermoforming technique using various thermoplastic materials. However, the thermoforming process alters the physical properties of the material, resulting in geometric inaccuracies, dimensional instability, reduced strength and lower wear resistance. [11] Recently, a clear photocurable resin for directly 3D printing aligners has become available in the market, allowing for fabrication of aligners with potentially improved precision, fit, and lighter, more constant force application than traditional thermoformed aligners [12, 13].

To improve the predictability of extrusive movements, the use of composite attachments and auxiliaries in the form of buttons and elastics has been proposed [9, 10]. However, patients often do not prefer their use as they may compromise the aesthetics and comfort [4]. Therefore, the purpose of the present study was to test different aligner materials and design features to achieve extrusion and evaluate the force and moment profiles.

## Materials and methods

**Fig. 1:**
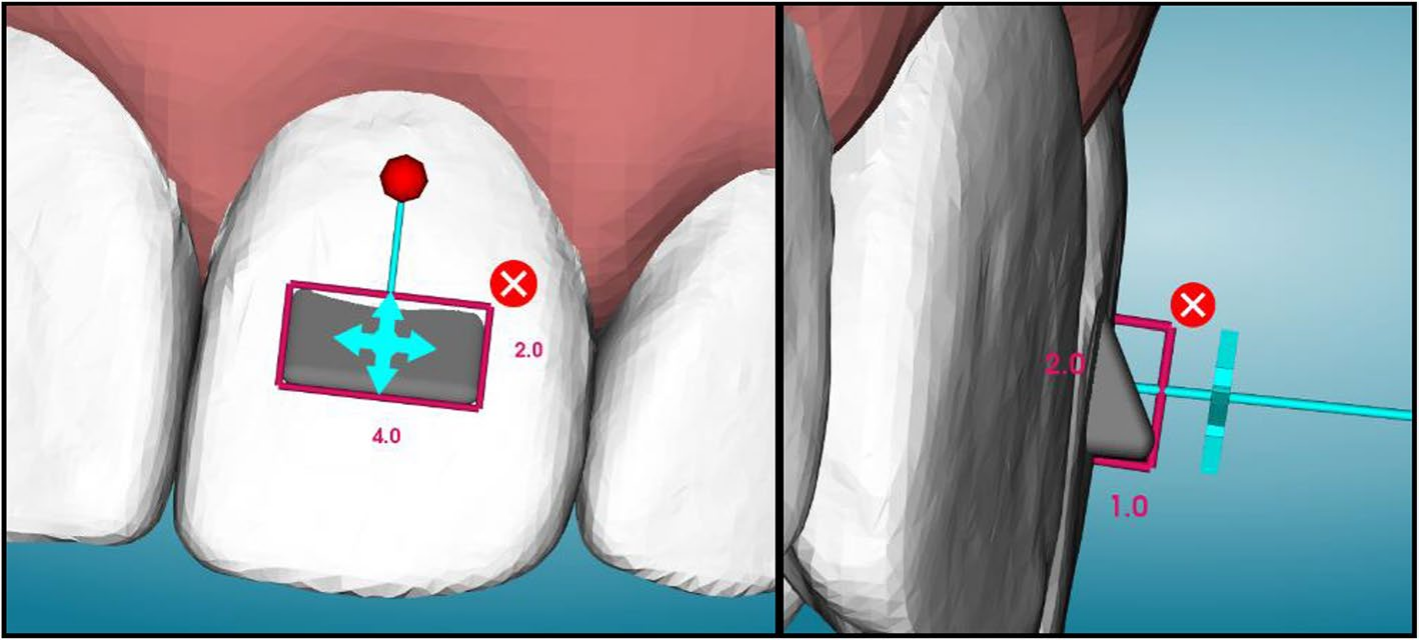
Attachment design and dimensions in uDesign 6.0

**Fig. 2:**
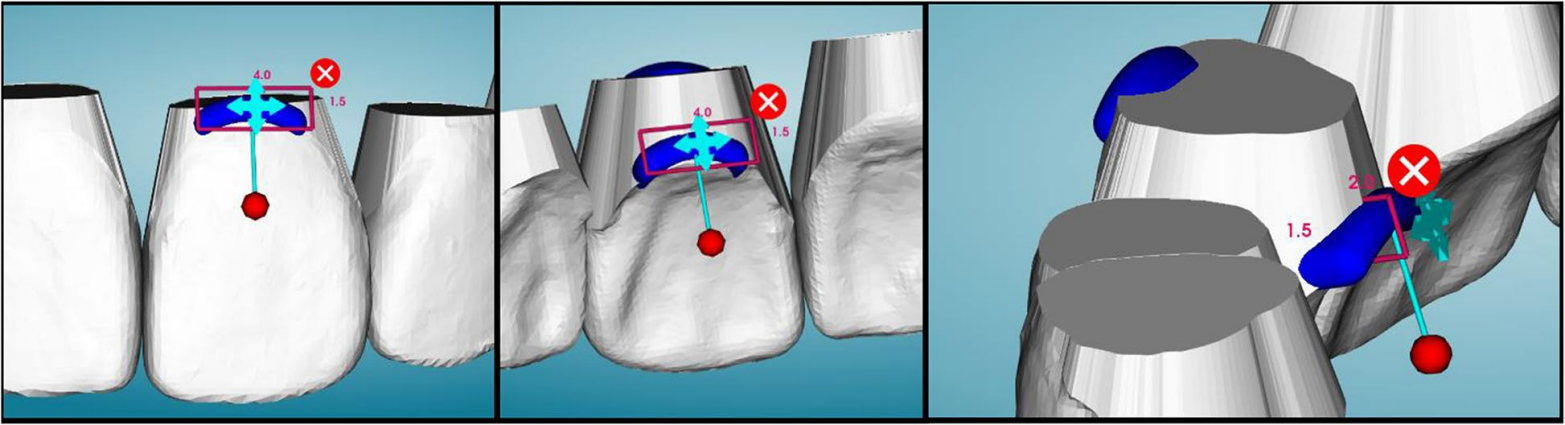
Pressure column design and dimensions in uDesign 6.0

Three different strategies for extrusion of a maxillary left central incisor (UL1) were tested using three different aligner materials for a total of nine different groups. Two of the aligner materials were thermoplastic foils: 0.030″ (0.76 mm) thickness ATMOS® (PET-G, American Orthodontics, Sheboygan, WI) and Zendura FLX® (Bay Materials LLC, Fremont, CA). The third material tested was the TC-85 clear photocurable resin (Graphy, Seoul, South Korea), manufactured specifically to 3D print clear aligners. For each material group, three extrusion strategies were employed: (1) No attachment or pressure columns, (2) Attachment only, and (3) Pressure columns only. Attachments were placed in the center of the clinical crown of the UL1 with dimensions of 4.0 mm in width, 2.0 mm in height, and 1.0 mm in depth (Fig. 1). The pressure columns were placed on facial and palatal surfaces, positioned as apically to the CEJ as the software would allow with dimensions of 4.0 mm in width, 1.5 mm in height, and 2.0 mm in depth (Fig. 2). The groups tested were as follows: ATMOS® control with no attachment or pressure columns (AC), ATMOS® with an attachment on UL1 (AA), ATMOS® with pressure columns on the UL1 (AP), Zendura FLX® control with no attachment or pressure columns (ZC), Zendura FLX® with an attachment on the UL1 (ZA), Zendura FLX® with pressure columns on UL1 (ZP), TC-85 control with no attachment or pressure columns (TC), TC-85 with an attachment on the UL1 (TA), and TC-85 with pressure columns (TP).

**Fig. 3:**
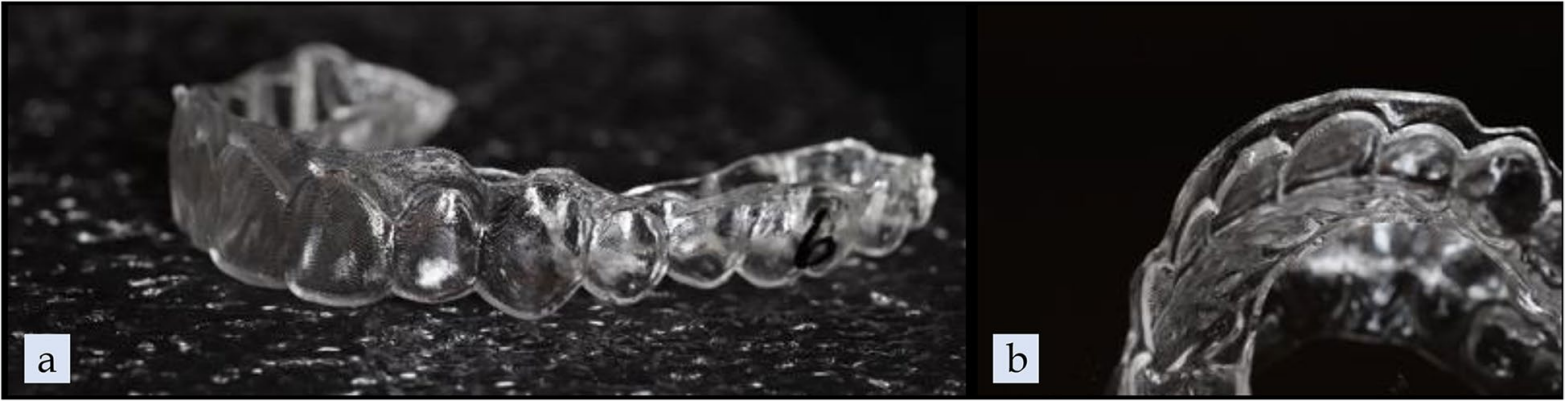
TC-85 pressure column group outer and internal views

**Fig. 4:**
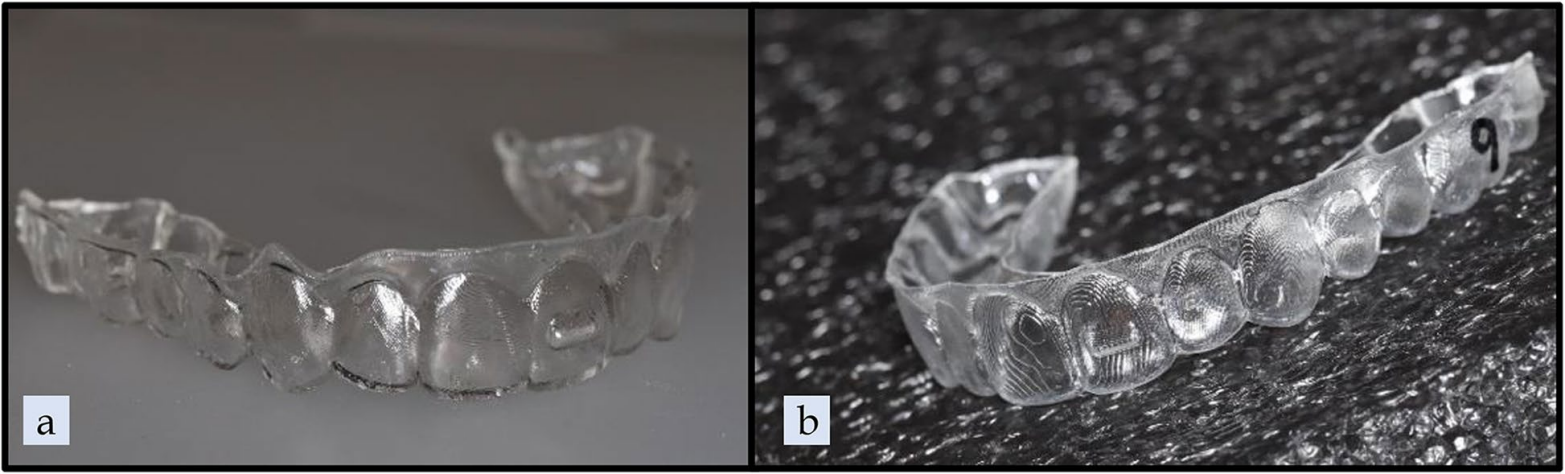
**a** TC-85 attachment group, **b** Thermoformed aligner attachment group

**Fig. 5:**
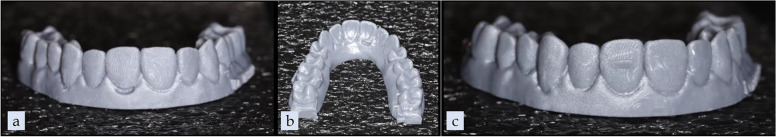
**a** Model for fabrication of thermoformed aligner pressure column group facial view, **b** Model for fabrication of thermoformed aligner pressure column group lingual view, **c** Model for fabrication of thermoformed aligner attachment group facial view

Within each group, ten aligners were tested, and 0.5 mm of extrusion was prescribed on the UL1. A custom version of uDesign® 6.0 software (uLab Systems Inc., Memphis, USA) was used to create the digital setup for extrusion along with attachment or pressure columns. For the 3D-printed aligner groups, the aligner shell files were exported from uDesign® 6.0 in STL file format and then imported into Uniz Maker (Uniz Technology LLC, San Diego, CA) to prepare for printing. The 3D-printed aligners were printed at 0.5-mm thickness with 0.03-mm offset and in 100-µm layers using SprintRay Pro 95 (SprintRay Inc., Los Angeles, CA). (Figs. 3, 4a) The same 3D printer was used to produce ten resin models for each thermoformed aligner group. The resin models were printed using Die and Model 2 Gray resin (SprintRay Inc., Los Angeles, CA) in 100-µm layers. A Biostarc vacuum forming machine (Scheu Dental GmbH, Iserlohn, Germany) was used to fabricate the thermoformed aligners following the manufacturer's instructions for the respective materials. (Figs. 4b, 5) For the pressure column groups, the gingival margin of each aligner was trimmed 1.5 mm apical to the facial and palatal pressure columns on the incisors; this was to ensure adequate material stiffness above the pressure columns.

**Fig. 6:**
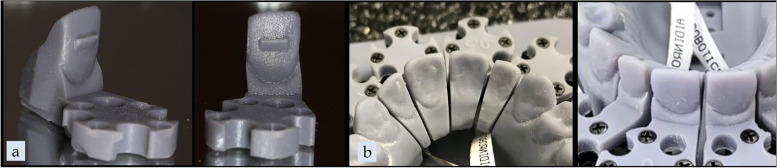
**a** 3D-printed tooth, **b** Facial and lingual gingival margin anatomy on printed tooth for sensor model

**Fig. 7:**
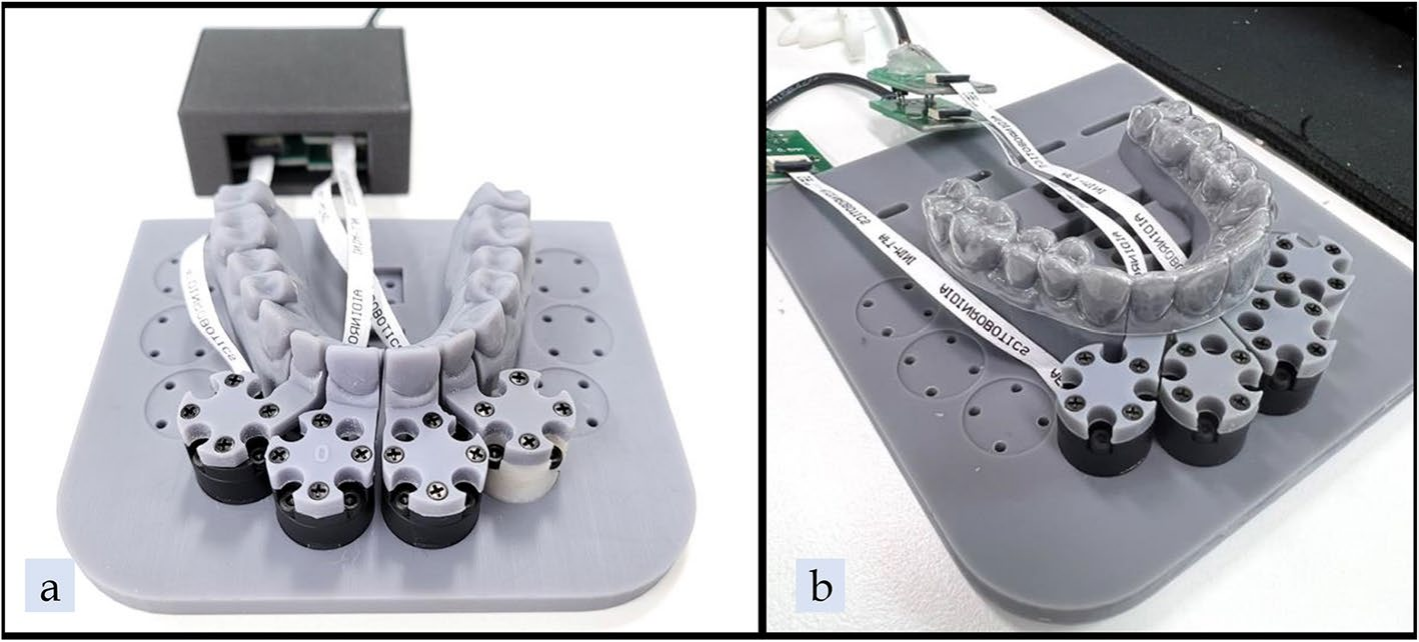
**a** 3-axis force and moment sensor apparatus, **b** Aligner seated on 3-axis force and moment sensor apparatus

**Fig. 8:**
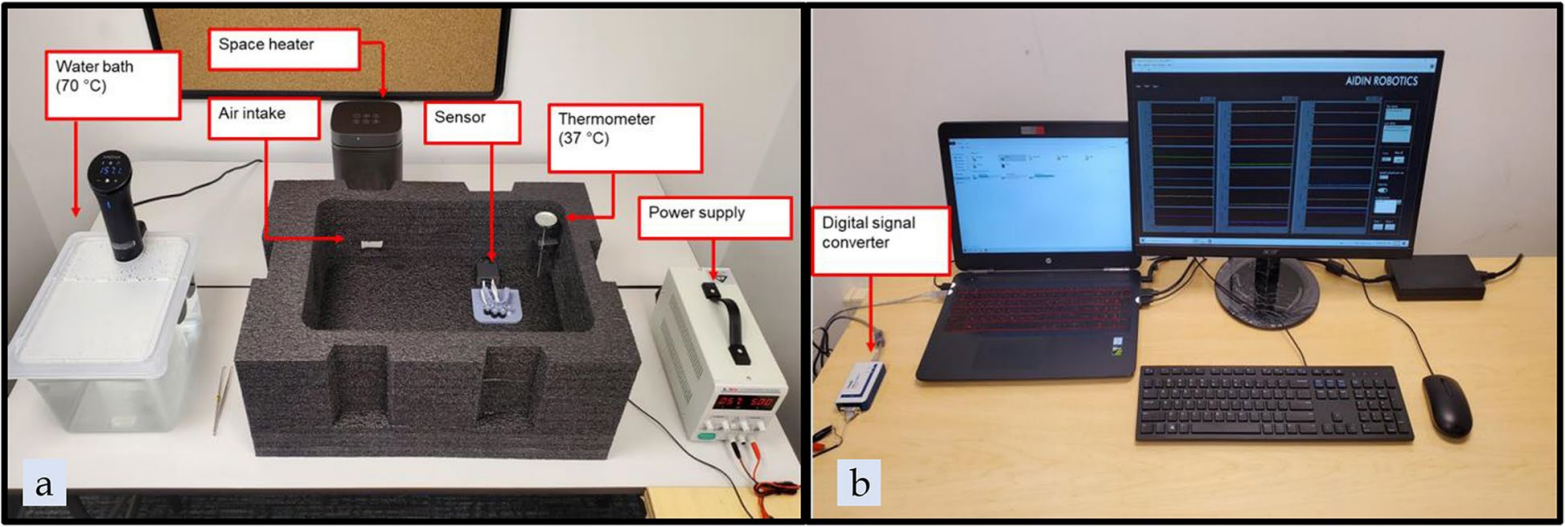
**a** Experimental environment setup, **b** Data collection and software setup

Following fabrication of the aligners, each aligner was fully seated onto a 3-axis force and moment sensor apparatus (Aidin Robotics, Anyang, South Korea), with sensors receiving force and moment data for the UL1 and the maxillary right central incisor (UR1) (Figs. 6, 7). The sensor apparatus was kept in a semi-enclosed chamber to simulate body temperature (37 °C) (Fig. 8). Prior to seating of each aligner, force and moment readings were initialized to zero to eliminate any external or mechanical influences on the readings. Each aligner was seated in an anterior to posterior direction from the incisors to the posterior dentition. Upon initial stabilization of the force and moment readings, the last 8.3 s of data was recorded for further analysis. For 3D-printed aligners, the aligners were first placed in a water bath of 69.4° C for 5 s and then fully seated onto the sensor apparatus and allowed to cool to 37 °C before the data was recorded. The water bath temperature of 69.4° C is the glass transition temperature of TC-85. By immersing the TC-85 aligners in warm water, force decay from deformation of the aligners can be reduced and the increased flexibility can ensure a better fit [12].

### Statistical analysis

All forces and moments were analyzed in reference to the estimated centers of resistance of the UL1 and UR1. Forces and moments for the three axes (x, y, and z) used in the study were summarized using means and standard deviations. For each tooth (UL1, UR1), the forces from each experimental condition (no attachment or pressure columns, attachment only, and pressure columns only) were compared for each aligner material separately. The forces by aligner material for each group respectively were also compared. Analysis of variance was used for the comparisons using PROC ANOVA with Bonferroni for multiple comparison (post-hoc) tests. All analyses were conducted by using SAS version 9.3 (SAS Inc., Cary, NC). Significance tests were performed by using 2-tailed hypothesis and the level of significance (α) was set to 0.05.

## Results

**Fig. 9:**
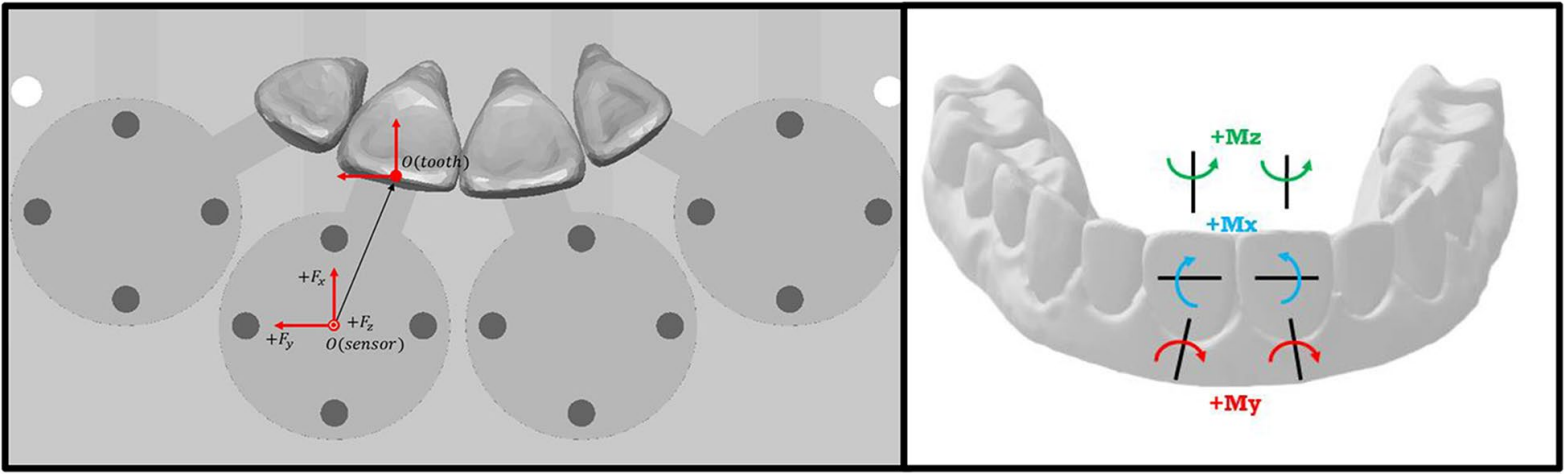
Conventions used for forces and moments in x,y,z axes

All force readings collected demonstrated statistically significant differences when compared by materials and when compared by groups, with a *P* value of < 0.001. Sign conventions from the sensor are represented in Fig. 9 and Table 1. A positive Fx represents lingual force, a positive Fy represents distal force for UL1 and mesial force for UR1, and a positive Fz represents extrusive force. Force values were measured in Newtons (N). A positive Mz represents mesial rotation for the UL1 and distal rotation for the UR1, a positive Mx represents mesial crown angulation for the UL1 and distal crown angulation for the UR1, and a positive My represents lingual inclination for both crowns. Negative values for any of the measurements represent movement in the opposite direction. Moment values were measured in Newton millimeters (Nmm).

**Table 1:** Force and moment sensor sign conversion

Component	Definition	Sign	UL1	UR1
Fx	Faciolingual	+ -	L F	L F
Fy	Mesiodistal	+ -	D M	M D
Fz	Occlusogingival	+ -	O G	O G
Mx	Angulation	+ -	M D	D M
My	Inclination	+ -	L F	L F
Mz	Rotation	+ -	M D	D M

### ATMOS® control with no attachment or pressure columns (AC)

**Fig. 10:**
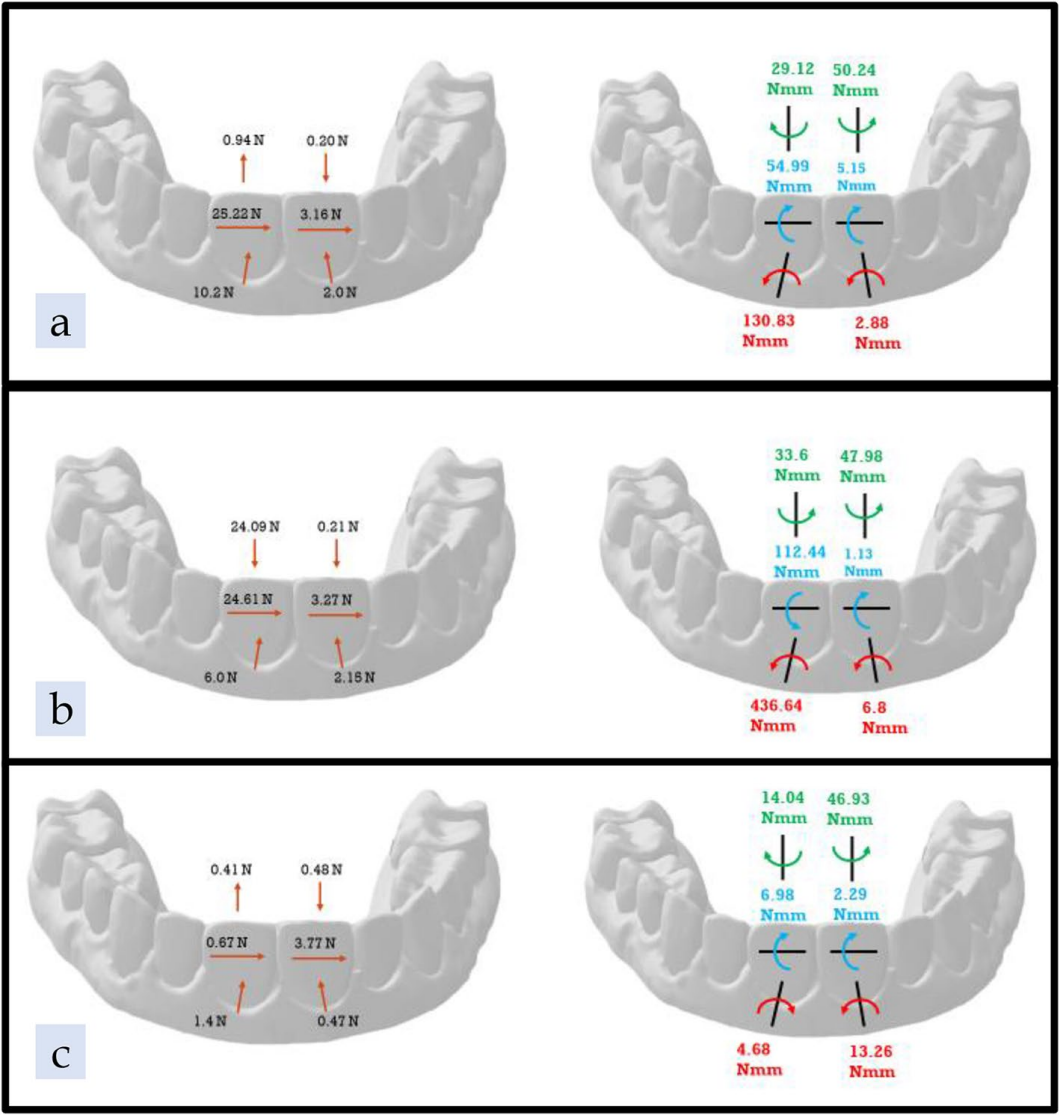
Results Diagram of the three tested groups; **a** ATMOS® group no pressure columns or attachments, **b** Zendura FLX® group no pressure columns or attachments, **c** TC-85 no pressure columns or attachments

In the AC group, the mean forces on the UL1 were 10.2 N of lingual force; 25.22 N of mesial force; and 0.94 N of extrusive force. The mean moments on the UL1 were 130.83 Nmm of distal angulation; 54.99 Nmm of lingual inclination; and 29.12 N of distal rotation (Fig. 10 a, Table 2). The mean forces on the UR1 were 2.00 N of lingual force; 3.16 N of distal force; and 0.20 N of intrusive force. Finally, the mean moments on the UR1 were 2.88 Nmm of mesial angulation; 5.15 Nmm of lingual inclination; and 50.24 Nmm of distal rotation (Fig. 10 a, Table 3).

**Table 2:** UL1 No attachment or pressure column groups comparison by material type

Force	ATMOS®		TC-85		Zendura FLX®		*P*-value^1^	*P*-value^2^	*P*-value^3^
	Mean	Std Dev	Mean	Std Dev	Mean	Std Dev			
Fx (N)	10.20	2.93	1.40	0.32	6.00	3.17	< 0.001	< 0.001	< 0.001
Fy (N)	-25.22	3.25	-0.67	0.28	-24.61	4.46	< 0.001	< 0.001	< 0.001
Fz (N)	0.94	16.91	0.41	0.17	-24.09	26.58	< 0.001	< 0.001	< 0.001
Mx (Nmm)	-130.83	211.30	4.68	2.09	-436.64	329.79	< 0.001	< 0.001	< 0.001
My (Nmm)	54.99	110.75	6.98	1.52	-112.44	173.27	< 0.001	< 0.001	< 0.001
Mz (Nmm)	-29.12	62.59	-14.04	2.93	33.60	41.49	< 0.001	< 0.001	< 0.001

*P*-value^1^ is a comparison between ATMOS® vs TC-85

*P*-value^2^ is a comparison between TC-85 vs Zendura FLX®

*P*-value^3^ is a comparison between ATMOS® vs Zendura FLX®

**Table 3:** UR1 No attachment or pressure column groups comparison by material type

Force	ATMOS®		TC-85		Zendura FLX®		*P*-value^1^	*P*-value^2^	*P*-value^3^
	Mean	Std Dev	Mean	Std Dev	Mean	Std Dev			
Fx (N)	2.00	0.41	0.47	0.18	2.15	0.98	< 0.001	< 0.001	< 0.001
Fy (N)	-3.16	2.88	-3.77	2.00	-3.27	5.39	< 0.001	< 0.001	< 0.001
Fz (N)	-0.20	1.00	-0.48	0.22	-0.21	0.65	< 0.001	< 0.001	< 0.001
Mx (Nmm)	-2.88	5.75	-13.26	5.04	-6.80	10.75	< 0.001	< 0.001	< 0.001
My (Nmm)	5.15	3.85	2.29	2.76	1.13	8.32	< 0.001	< 0.001	< 0.001
Mz (Nmm)	50.24	36.51	46.93	23.16	47.98	58.73	< 0.001	< 0.001	< 0.001

*P*-value^1^ is a comparison between ATMOS® vs TC-85

*P*-value^2^ is a comparison between TC-85 vs Zendura FLX®

*P*-value^3^ is a comparison between ATMOS® vs Zendura FLX®

### Zendura FLX® control with no attachment or pressure columns (ZC)

In the ZC group, the UL1 measured 6.00 N of lingual force, which was lower than that in the AC group; 24.61 N of mesial force; and 24.09 N of intrusive force, which was opposite in direction to the AC group. Mean moments on the UL1 were 436.64 Nmm of distal angulation; 112.44 Nmm of facial inclination; and 33.6 Nmm of mesial rotation. The crown inclination and rotational moments were opposite in direction to the moments generated by the AC group (Fig. 10 b, Table 2). The UR1 measured 2.15 N of lingual force; 3.27 N of distal force; 0.21 N of intrusive force; 6.8 Nmm of mesial angulation; 1.13 Nmm of lingual inclination; and 47.98 Nmm of distal rotation. These values were comparable to the values from the AC group on the UR1, except for crown angulation and inclination moments (Fig. 10 b, Table 3).

### TC-85 control with no attachments or pressure columns (TC)

In the TC group, the UL1 measured 1.40 N of lingual force; 0.67 N of mesial force; 0.41 N of extrusive force; 4.68 Nmm of mesial crown angulation; 6.98 Nmm of lingual inclination; and 14.04 Nmm of distal rotation (Fig. 10 c, Table 2). The forces and moments measured by the UR1 were 0.47 N of lingual force; 3.77 N of distal force; 0.48 N of intrusive force; 13.26 Nmm of mesial crown angulation; 2.29 Nmm of lingual inclination; and 46.93 Nmm of distal rotation (Fig. 10 c, Table 3). Significantly lower forces and moments were exerted by the TC group in comparison to the AC and ZC groups.

### ATMOS® with attachments only (AA)

**Fig. 11:**
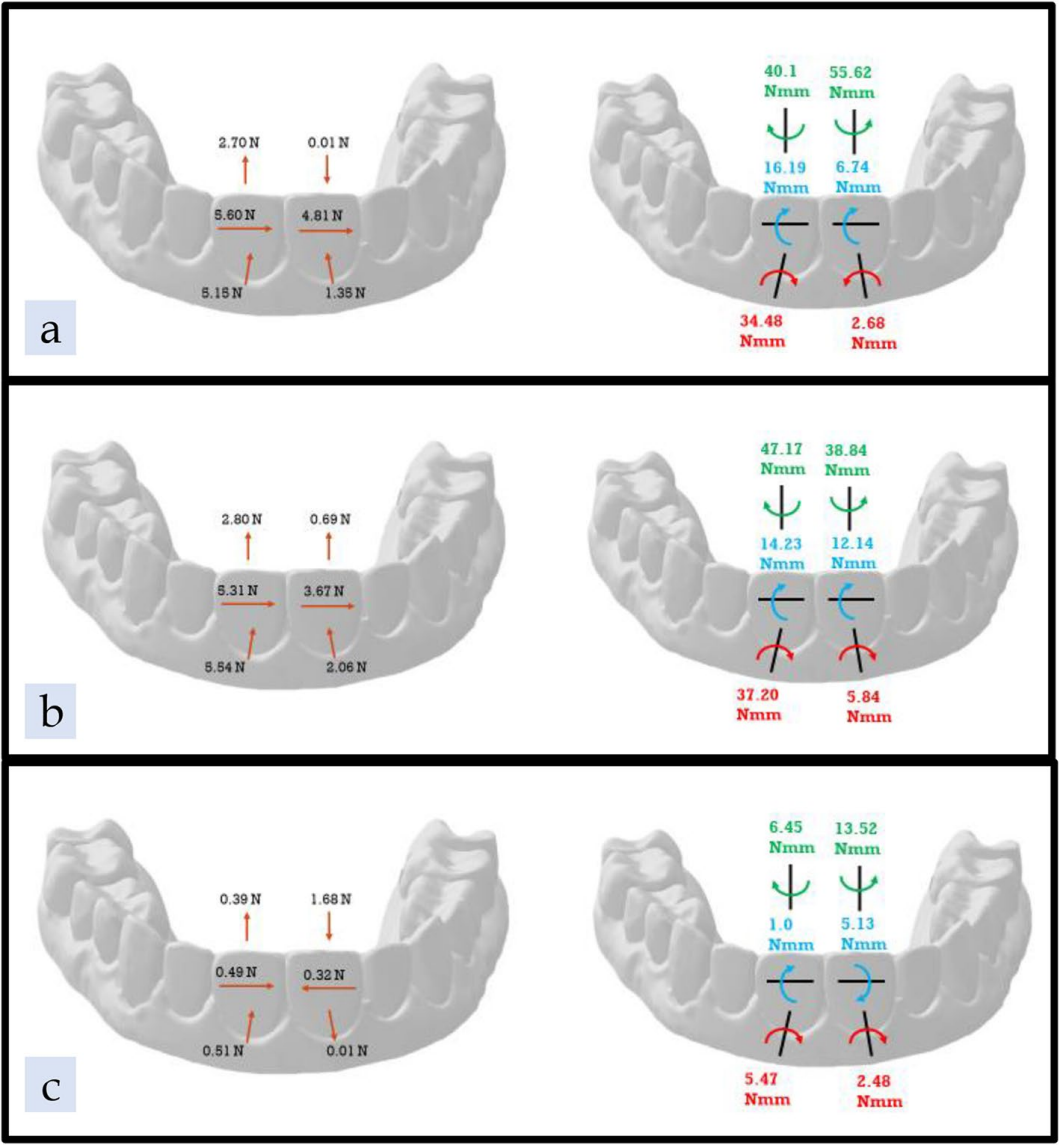
Results Diagram of the three tested groups; **a** ATMOS® Attachment group, **b** Zendura FLX® Attachment group, **c** TC-85 Attachment group

In the AA group, the mean forces and moments on the UL1 were 5.15 N of lingual force; 5.60 N of mesial force; 2.70 N of extrusive force; 34.48 Nmm of mesial angulation; 16.19 Nmm of lingual inclination; and 40.1 Nmm of distal rotation (Fig. 11 a, Table 4). The forces and moments on the UR1 were 1.35 N of lingual force; 4.81 N of distal force; 0.01 N of intrusive force; 2.68 Nmm of mesial angulation; 6.74 Nmm of lingual inclination; and 55.62 Nmm of distal rotation (Fig. 11 a, Table 5).

**Table 4:** UL1 Attachment groups comparison by material type

Force	ATMOS®		TC-85		Zendura FLX®		*P*-value^1^	*P*-value^2^	*P*-value^3^
	Mean	Std Dev	Mean	Std Dev	Mean	Std Dev			
Fx (N)	5.15	0.39	0.51	0.36	5.54	0.32	< 0.001	< 0.001	< 0.001
Fy (N)	-5.61	0.98	-0.49	0.39	-5.31	1.03	< 0.001	< 0.001	< 0.001
Fz (N)	2.70	0.67	0.39	0.29	2.80	0.28	< 0.001	< 0.001	< 0.001
Mx (Nmm)	34.48	8.39	5.47	2.70	37.20	4.49	< 0.001	< 0.001	< 0.001
My (Nmm)	16.19	5.81	1.00	2.77	14.23	5.22	< 0.001	< 0.001	< 0.001
Mz (Nmm)	-40.10	7.62	-6.45	6.83	-47.17	9.71	< 0.001	< 0.001	< 0.001

*P*-value^1^ is a comparison between ATMOS® vs TC-85

*P*-value^2^ is a comparison between TC-85 vs Zendura FLX®

*P*-value^3^ is a comparison between ATMOS® vs Zendura FLX®

**Table 5:** UR1 Attachment groups comparison by material type

Force	ATMOS®		TC-85		Zendura FLX®		*P*-value^1^	*P*-value^2^	*P*-value^3^
	Mean	Std Dev	Mean	Std Dev	Mean	Std Dev			
Fx (N)	1.35	0.45	-0.01	0.22	2.06	0.41	< 0.001	< 0.001	< 0.001
Fy (N)	-4.81	1.83	0.32	1.24	-3.67	0.60	< 0.001	< 0.001	< 0.001
Fz (N)	-0.01	0.72	-1.68	0.25	0.69	0.98	< 0.001	< 0.001	< 0.001
Mx (Nmm)	-2.68	6.90	2.48	2.82	5.84	5.61	< 0.001	< 0.001	< 0.001
My (Nmm)	6.74	5.75	-5.13	3.17	12.14	8.50	< 0.001	< 0.001	< 0.001
Mz (Nmm)	55.62	20.37	13.52	15.14	38.84	6.86	< 0.001	< 0.001	< 0.001

*P*-value^1^ is a comparison between ATMOS® vs TC-85

*P*-value^2^ is a comparison between TC-85 vs Zendura FLX®

*P*-value^3^ is a comparison between ATMOS® vs Zendura FLX®

### Zendura FLX® with attachments only (ZA)

In the ZA group, the mean forces and moments on the UL1 were 5.54 N of lingual force; 5.31 N of mesial force; 2.80 N of extrusive force; 37.2 Nmm of mesial angulation; 14.23 Nmm of lingual inclination; and 47.17 Nmm of distal rotation (Fig. 11 b, Table 4). The mean forces and moments on the UR1 in the ZA group were 2.06 N of lingual force; 3.67 N of distal force; 0.69 N of extrusive force; 5.84 Nmm of distal angulation; 12.14 Nmm of lingual inclination; and 38.84 Nmm of distal rotation (Fig. 11 b, Table 5). With the UL1, the forces and moments were comparable for both the AA and ZA groups. With the UR1, the groups differed in the directions of vertical forces and crown angulation moments.

### TC-85 with attachments only (TA)

In the TA group, the mean forces and moments on the UL1 were 0.51 N of lingual force; 0.49 N of mesial force; 0.39 N of extrusive force; 5.47 Nmm of mesial crown angulation; 1.00 Nmm of lingual inclination; and 6.45 Nmm of distal rotation (Fig. 11 c, Table 4). The mean forces and moments on the UR1 were 0.01 N of buccal force; 0.32 N of mesial force; 1.68 N of intrusive force; 2.48 Nmm of distal crown angulation; 5.13 Nmm of facial crown inclination; and 13.52 Nmm of distal rotation (Fig. 11 c, Table 5). With the UL1, the forces and moments of the TA group were substantially lower than those of the thermoformed aligner (AA and ZA) groups. With the UR1, when compared to the thermoformed aligner groups, the TA group differed in the directions of faciolingual force, mesiodistal force and faciolingual inclination.

### ATMOS® with pressure columns only (AP)

**Fig. 12:**
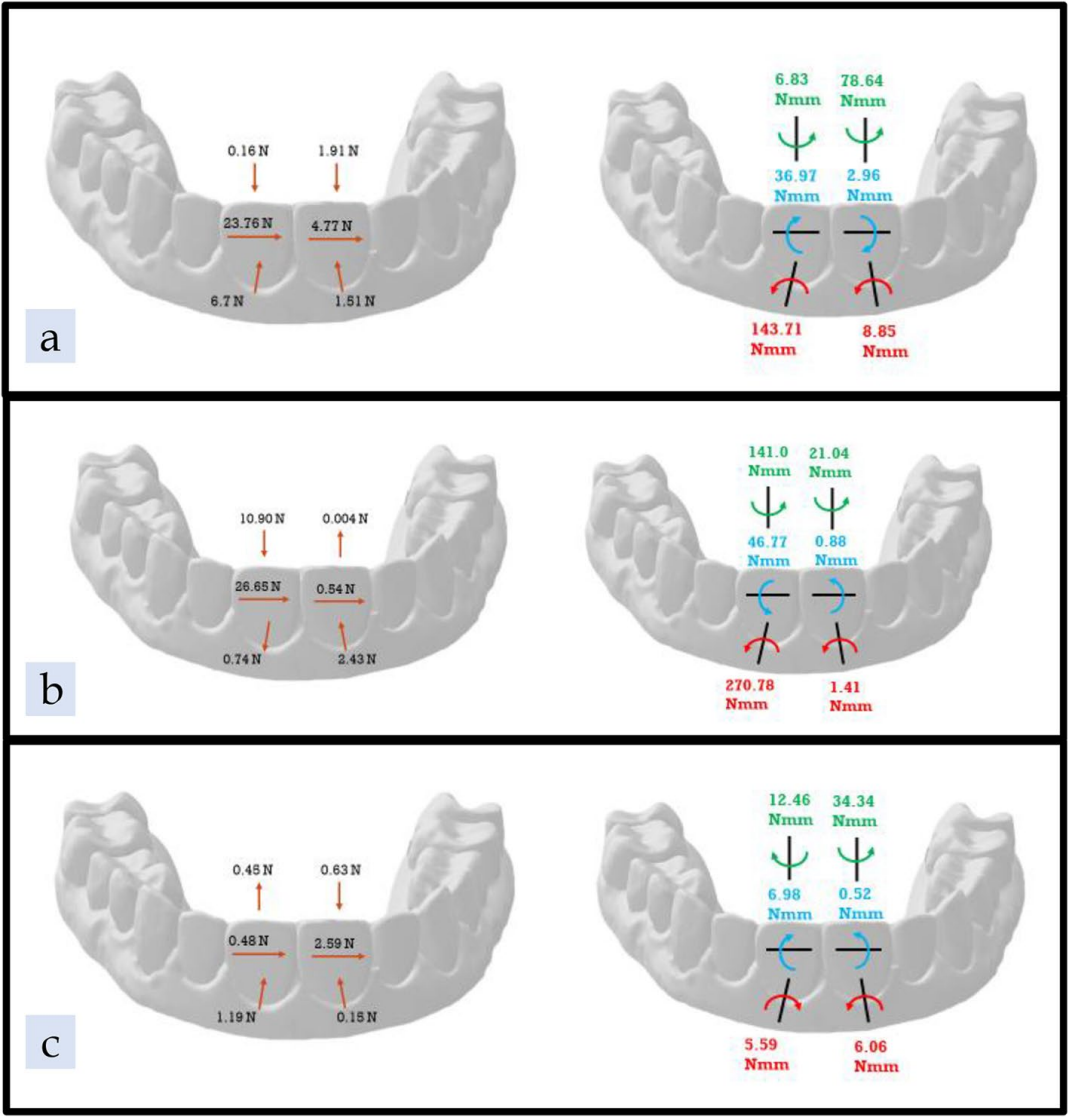
Results Diagram of the three tested groups; **a** ATMOS® pressure columns group, **b** Zendura FLX® pressure columns group, **c** TC-85 pressure columns group

In the AP group, the mean forces and moments on the UL1 were 6.7 N of lingual force; 23.76 N of mesial force; 0.16 N of intrusive force; 143.71 Nmm of distal angulation; 36.97 Nmm of lingual inclination; and 6.83 Nmm of mesial rotation (Fig. 12 a, Table 6). The mean forces and moments on the UR1 were 1.51 N of lingual force; 4.77 N of distal force; 1.91 N of intrusive force; 8.85 Nmm of mesial angulation; 2.96 Nmm of buccal inclination; and 78.64 Nmm of distal rotation. The AP group was the only pressure column group exhibiting a mean moment with buccal inclination on the UR1 (Fig. 12 a, Table 7).

**Table 6:** UL1 Pressure column groups comparison by material type

Force	ATMOS®		TC-85		Zendura FLX®		*P*-value^1^	*P*-value^2^	*P*-value^3^
	Mean	Std Dev	Mean	Std Dev	Mean	Std Dev			
Fx (N)	6.71	4.59	1.19	0.27	-0.74	4.92	< 0.001	< 0.001	< 0.001
Fy (N)	-23.76	2.19	-0.48	0.36	-26.65	4.61	< 0.001	< 0.001	< 0.001
Fz (N)	-0.16	16.34	0.45	0.16	-10.90	26.50	< 0.001	< 0.001	< 0.001
Mx (Nmm)	-143.71	204.61	5.59	2.19	-270.78	320.89	< 0.001	< 0.001	< 0.001
My (Nmm)	36.97	112.46	6.98	1.61	-46.77	171.76	< 0.001	< 0.001	< 0.001
Mz (Nmm)	6.83	74.28	-12.46	3.20	141.00	60.40	< 0.001	< 0.001	< 0.001

*P*-value^1^ is a comparison between ATMOS® vs TC-85

*P*-value^2^ is a comparison between TC-85 vs Zendura FLX®

*P*-value^3^ is a comparison between ATMOS® vs Zendura FLX®

**Table 7:** UR1 Pressure column groups comparison by material type

Force	ATMOS®		TC-85		Zendura FLX®		*P*-value^1^	*P*-value^2^	*P*-value^3^
	Mean	Std Dev	Mean	Std Dev	Mean	Std Dev			
Fx (N)	1.51	1.28	0.15	0.16	2.43	0.36	< 0.001	< 0.001	< 0.001
Fy (N)	-4.77	3.71	-2.59	1.20	-0.54	1.66	< 0.001	< 0.001	< 0.001
Fz (N)	1.91	2.71	-0.63	0.32	0.00	0.64	< 0.001	< 0.001	< 0.001
Mx (Nmm)	-8.85	12.15	-6.06	5.90	-1.41	6.63	< 0.001	< 0.001	< 0.001
My (Nmm)	-2.96	17.53	0.52	1.95	0.88	4.80	< 0.001	< 0.001	< 0.001
Mz (Nmm)	78.64	51.26	34.34	13.30	21.04	19.66	< 0.001	< 0.001	< 0.001

*P*-value^1^ is a comparison between ATMOS® vs TC-85

*P*-value^2^ is a comparison between TC-85 vs Zendura FLX®

*P*-value^3^ is a comparison between ATMOS® vs Zendura FLX®

### Zendura FLX® with pressure columns only (ZP)

In the ZP group, the mean forces and moments on the UL1 were 0.74 N of buccal force, which was opposite in direction to the AP group; 26.65 N of mesial force; 10.90 N of intrusive force; 270.78 Nmm of distal crown angulation; 46.77 Nmm of facial crown inclination; and 141.0 Nmm of mesial rotation (Fig. 12 b, Table 6). The magnitudes of moments on the UL1 were higher in the ZP group relative to the AP group. The mean forces and moments on the UR1 were 2.43 N of lingual force; 0.54 N of distal force; 0.004 N of extrusive force which was opposite in direction to the AP and TP groups; 1.41 Nmm of mesial crown angulation; 0.88 Nmm of lingual inclination; and 21.04 Nmm of distal rotation (Fig. 12 b, Table 7).

### TC-85 with pressure columns only (TP)

In the TP group, the mean forces and moments on the UL1 were 1.19 N of lingual force; 0.48 N of mesial force; 0.45 N of extrusive force; 5.59 Nmm of mesial crown angulation; 6.98 Nmm of lingual inclination; and 12.46 Nmm of distal rotation (Fig. 12 c, Table 6). Among the pressure column groups, the TP group was the only group showing extrusion on the UL1. The mean forces and moments on the UR1 were 0.15 N of lingual force; 2.59 N of distal force; 0.63 N of intrusive force; 6.06 Nmm of mesial crown angulation; 0.52 Nmm of lingual inclination; and 34.34 Nmm of distal rotation (Fig. 12 c, Table 7).

**Fig. 13:**
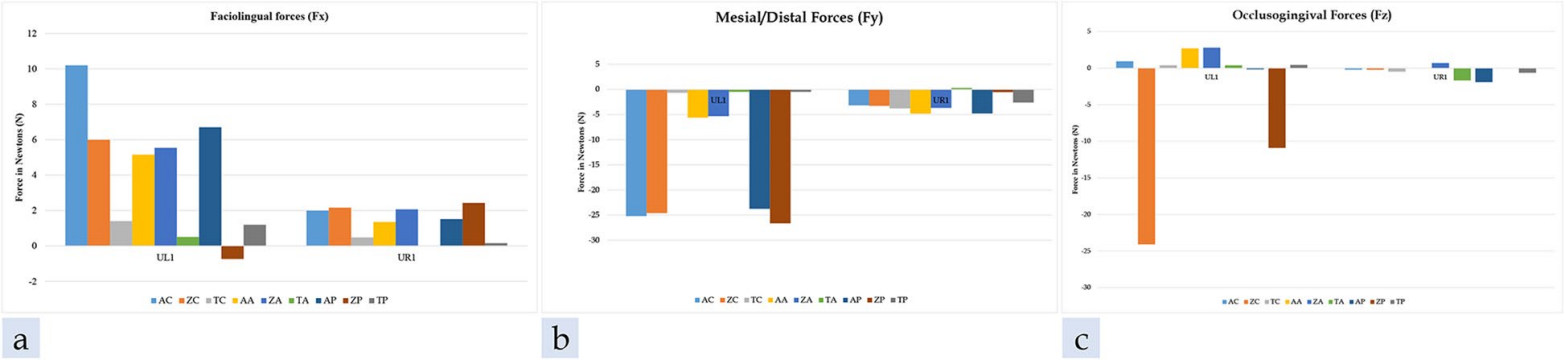
**a** Faciolingual forces on UL1 and UR1, **b** Mesiodistal forces on UL1 and UR1, **c** Occlusogingival forces on UL1 and UR1 by the three different tested materials

**Fig. 14:**
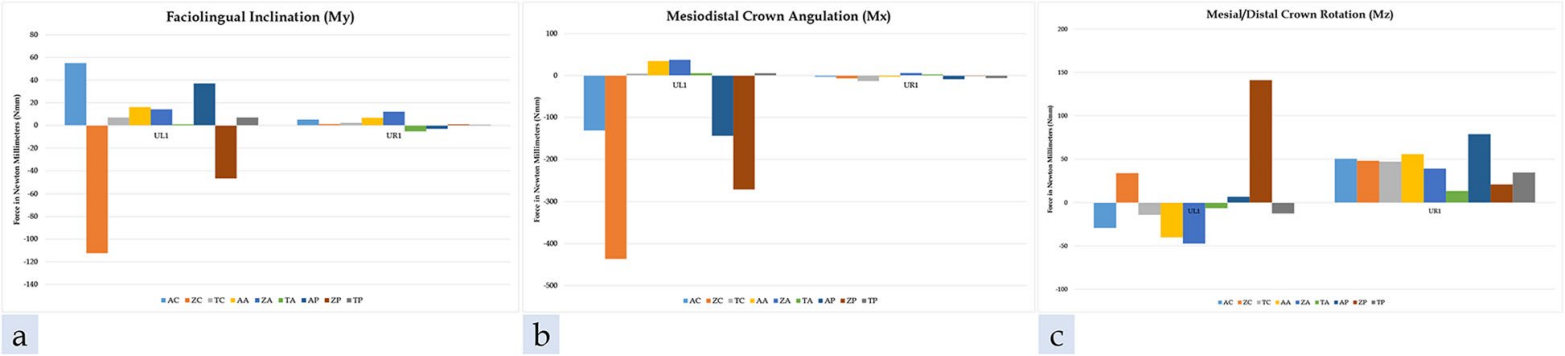
**a** Faciolingual inclination moments on UL1 and UR1, **b** Mesiodistal crown angulation moments on UL1 and UR1, **c** Mesiodistal crown rotation moments on UL1 and UR1 by the three different tested materials

The mean forces and moments exerted on the UL1 were tabulated by each material type and the three extrusion strategies in Tables 8, 9 and 10; the mean forces and moments exerted on the UR1 were tabulated by each material type and the three extrusion strategies in Tables 11, 12 and 13. The mean forces and moments generated on the UL1 and UR1 are presented in graph format for all nine groups in Figs. 13, 14.

**Table 8:** UL1 Comparison of ATMOS® groups

Force	No attachment		Attachment		Pressure columns		*P*-value^a^	*P*-value^b^	*P*-value^c^
	Mean	Std Dev	Mean	Std Dev	Mean	Std Dev			
Fx (N)	10.20	2.93	5.15	0.39	6.71	4.59	< 0.001	< 0.001	< 0.001
Fy (N)	-25.22	3.25	-5.61	0.98	-23.76	2.19	< 0.001	< 0.001	< 0.001
Fz (N)	0.94	16.91	2.70	0.67	-0.16	16.34	< 0.001	< 0.001	< 0.001
Mx (Nmm)	-130.83	211.30	34.48	8.39	-143.71	204.61	< 0.001	< 0.001	< 0.001
My (Nmm)	54.99	110.75	16.19	5.81	36.97	112.46	< 0.001	< 0.001	< 0.001
Mz (Nmm)	-29.12	62.59	-40.10	7.62	6.83	74.28	< 0.001	< 0.001	< 0.001

*P*-value^a^ is a comparison between control vs attachment

*p*-value^b^ is a comparison between attachment vs pressure columns

*P*-value^c^ is a comparison between control vs pressure columns

**Table 9:** UL1 Comparison of Zendura FLX® groups

Force	No attachment		Attachment		Pressure columns		*P*-value^a^	*P*-value^b^	*P*-value^c^
	Mean	Std Dev	Mean	Std Dev	Mean	Std Dev			
Fx (N)	6.00	3.17	5.54	0.32	-0.74	4.92	< 0.001	< 0.001	< 0.001
Fy (N)	-24.61	4.46	-5.31	1.03	-26.65	4.61	< 0.001	< 0.001	< 0.001
Fz (N)	24.09	26.58	2.80	0.28	-10.90	26.50	< 0.001	< 0.001	< 0.001
Mx (Nmm)	-436.64	329.79	37.20	4.49	-270.78	320.89	< 0.001	< 0.001	< 0.001
My (Nmm)	-112.44	173.27	14.23	5.22	-46.77	171.76	< 0.001	< 0.001	< 0.001
Mz (Nmm)	33.60	41.49	-47.17	9.71	141.00	60.40	< 0.001	< 0.001	< 0.001

*P*-value^a^ is a comparison between control vs attachment

*p*-value^b^ is a comparison between attachment vs pressure columns

*P*-value^c^ is a comparison between control vs pressure columns

**Table 10:** UL1 Comparison of TC-85 groups

Force	No attachment		Attachment		Pressure columns		*P*-value^a^	*P*-value^b^	*P*-value^c^
	Mean	Std Dev	Mean	Std Dev	Mean	Std Dev			
Fx (N)	1.40	0.32	0.51	0.36	1.19	0.27	< 0.001	< 0.001	< 0.001
Fy (N)	-0.67	0.28	-0.49	0.39	-0.48	0.36	< 0.001	< 0.001	< 0.001
Fz (N)	0.41	0.17	0.39	0.29	0.45	0.16	< 0.001	< 0.001	< 0.001
Mx (Nmm)	4.68	2.09	5.47	2.70	5.59	2.19	< 0.001	< 0.001	< 0.001
My (Nmm)	6.98	1.52	1.00	2.77	6.98	1.61	< 0.001	< 0.001	< 0.001
Mz (Nmm)	-14.04	2.93	-6.45	6.83	-12.46	3.20	< 0.001	< 0.001	< 0.001

*P*-value^a^ is a comparison between control vs attachment

*p*-value^b^ is a comparison between attachment vs pressure columns

*P*-value^c^ is a comparison between control vs pressure columns

**Table 11:** UR1 Comparison of ATMOS® groups

Force	No attachment		Attachment		Pressure columns		*P*-value^a^	*P*-value^b^	*P*-value^c^
	Mean	Std Dev	Mean	Std Dev	Mean	Std Dev			
Fx (N)	2.00	0.41	1.35	0.45	1.51	1.28	< 0.001	< 0.001	< 0.001
Fy (N)	-3.16	2.88	-4.81	1.83	-4.77	3.71	< 0.001	< 0.001	< 0.001
Fz (N)	-0.20	1.00	-0.01	0.72	-1.91	2.71	< 0.001	< 0.001	< 0.001
Mx (Nmm)	-2.88	5.75	-2.68	6.90	-8.85	12.15	< 0.001	< 0.001	< 0.001
My (Nmm)	5.15	3.85	6.74	5.75	-2.96	17.53	< 0.001	< 0.001	< 0.001
Mz (Nmm)	50.24	36.51	55.62	20.37	78.64	51.26	< 0.001	< 0.001	< 0.001

*P*-value^a^ is a comparison between control vs attachment

*p*-value^b^ is a comparison between attachment vs pressure columns

*P*-value^c^ is a comparison between control vs pressure columns

**Table 12:** UR1 Comparison of Zendura FLX® groups

Force	No attachment		Attachment		Pressure columns		*P*-value^a^	*P*-value^b^	*P*-value^c^
	Mean	Std Dev	Mean	Std Dev	Mean	Std Dev			
Fx (N)	2.15	0.98	2.06	0.41	2.43	0.36	< 0.001	< 0.001	< 0.001
Fy (N)	-3.27	5.39	-3.67	0.60	-0.54	1.66	< 0.001	< 0.001	< 0.001
Fz (N)	-0.21	0.65	0.69	0.98	0.00	0.64	< 0.001	< 0.001	< 0.001
Mx (Nmm)	-6.80	10.75	5.84	5.61	-1.41	6.63	< 0.001	< 0.001	< 0.001
My (Nmm)	1.13	8.32	12.14	8.50	0.88	4.80	< 0.001	< 0.001	< 0.001
Mz (Nmm)	47.98	58.73	38.84	6.86	21.04	19.66	< 0.001	< 0.001	< 0.001

*P*-value^a^ is a comparison between control vs attachment

*p*-value^b^ is a comparison between attachment vs pressure columns

*P*-value^c^ is a comparison between control vs pressure columns

**Table 13:** UR1 Comparison of TC-85 groups

Force	No attachment		Attachment		Pressure columns		*P*-value^a^	*P*-value^b^	*P*-value^c^
	Mean	Std Dev	Mean	Std Dev	Mean	Std Dev			
Fx (N)	0.47	0.18	-0.01	0.22	0.15	0.16	< 0.001	< 0.001	< 0.001
Fy (N)	-3.77	2.00	0.32	1.24	-2.59	1.20	< 0.001	< 0.001	< 0.001
Fz (N)	-0.48	0.22	-1.68	0.25	-0.63	0.32	< 0.001	< 0.001	< 0.001
Mx (Nmm)	-13.26	5.04	2.48	2.82	-6.06	5.90	< 0.001	< 0.001	< 0.001
My (Nmm)	2.29	2.76	-5.13	3.17	0.52	1.95	< 0.001	< 0.001	< 0.001
Mz (Nmm)	46.93	23.16	13.52	15.14	34.34	13.30	< 0.001	< 0.001	< 0.001

*P*-value^a^ is a comparison between control vs attachment

*p*-value^b^ is a comparison between attachment vs pressure columns

*P*-value^c^ is a comparison between control vs pressure columns

## Discussion

Clear aligner therapy is an increasingly popular treatment modality due to patient interest in esthetic and comfortable alternatives to conventional fixed appliances [1, 4]. However, the overall predictability of tooth movements using clear aligners is lower than that of fixed appliances in spite of numerous advances in materials science and digital technology. Extrusion of anterior teeth is among the least accurate movements to achieve using clear aligners [6–8]. Clinicians have attempted to improve the predictability of extrusive movements by modifying the force system acting on the tooth using adjuncts such as composite attachments and modifying the geometric shape of the aligners [9, 10]. Therefore, the aim of the present study was to evaluate the force systems produced by various extrusion strategies utilizing different material types and adjunctive design features on the aligners.

### Extrusion with clear aligners

All three attachment groups in the study (AA, ZA, TA), regardless of the aligner material, exerted a mean extrusive force on the UL1. Although there is uncertainty in the literature regarding the effectiveness of composite attachments in improving the predictability of extrusion [14, 15], the present findings suggest that attachments aid in applying consistent extrusive forces on the maxillary central incisor. The additional surface geometry from the attachment may enhance retention of the aligner and improve force delivery.

In the clinical setting, the need for attachments to improve predictability may need to be balanced with the patient’s desire for a more esthetic appliance by avoiding the use of attachments on the anterior dentition. In the present study, aligners were designed with pressure columns, or indentations on the intaglio surface along the cervical margins of the UL1. Among the three material types, the 3D-printed aligner group (TP) showed mean extrusive force on the UL1; thermoformed aligner groups with pressure columns (AP, ZP) showed mean intrusive forces.

In the absence of attachments or pressure columns, extrusive force on the UL1 was noted with the AC and TC groups. Interestingly, the ZC group did not display extrusive force on the UL1; rather, there was a high level of intrusive force. These findings highlight the unpredictable nature of force systems created by clear aligners, especially without the use of adjuncts.

### Unplanned forces and moments

When 0.5 mm of extrusive movement was prescribed to the UL1, the UL1 and UR1 experienced a mean lingual force; this was true for all groups except for the ZP group with the UL1 and the TA group with the UR1. In general, the majority of the groups also experienced a moment to tip the crown lingually, with the exception of two groups for the UL1 (ZC, ZP) and two groups for the UR1 (TA, AP). The presence of lingual force during extrusive movement is consistent with previous findings in the literature [16, 17].

Interestingly, in all groups, the UL1 received a mean force in the mesial direction. Likewise, the UR1 also received a mean force in the mesial direction in all groups. The magnitudes of the forces were generally lower with the TC-85 groups than the ATMOS and Zendura FLX groups. Regardless of material type, all three attachment groups (AA, ZA, TA) resulted in a mesial crown angulation moment on the UL1. In the present study, the attachment on the UL1 was oriented as horizontally as possible perpendicular to the direction of extrusion, in accordance to Rossini et al. (2021) who reported that a rectangular horizontal attachment on the buccal or palatal surface of the maxillary incisors resulted in the most efficient force system with minimal aligner deformation [17].

For the UL1, there was a moment to rotate the crown distally in six groups (AC, TC, AA, ZA, TA, TP), and a moment to rotate the crown mesially in three groups (ZC, AP, ZP). For the UR1, there was a moment to rotate the crown distally in all nine groups.

Although the UL1 was planned for bodily extrusion only, a complex force system was present with forces and moments in all three dimensions not only on the UL1 but also on the adjacent UR1. The magnitudes of the unplanned forces and moments measured were sometimes several times higher than conventionally accepted values for physiologic tooth movement. These results point to the complexity and lack of predictability of clear aligner biomechanics.

Additionally, the variability of the mean forces and moments was higher with thermoformed aligners versus 3D-printed aligners. In the clinical setting, this may imply a high level of unpredictability in force systems during clear aligner therapy when using conventional thermoplastic materials. With 3D-printed aligners, the inherent advantages of the 3D-printing process ensure higher dimensional accuracy and consequently better fitting aligners; this is reflected by the lower variation in the mean force and moment values across the study.

### Excessive force levels

A recent systematic review concluded that the ideal force level for orthodontic tooth movement is between 0.5–1.0 N [18]. In the present study, with the thermoformed aligner groups, the mean force levels exceeded over 20 times the physiologic range. The TC-85 3D-printed aligner groups demonstrated lower, more biologically harmonious force levels, although some groups still exceeded force levels of 4—5 N.

A potential explanation for the extreme force levels and unpredictable forces and moments reported in the present study may be the amount of tooth movement prescribed on the UL1. A 0.5 mm of extrusive movement may have exceeded the elastic range of the plastic, possibly leading to a lack of fit between the aligner and the dentition, and thus creating undesirable and unexpected force systems. It is possible that extrusion in smaller increments may result in lower force levels and more predictable force systems. Future studies should evaluate the role of the amount of planned tooth movement on the resulting force system during extrusion.

### Limitations

There are several limitations with the in vitro experimental design, namely the lack of simulated saliva, periodontal ligaments, and masticatory forces. Moreover, the present study investigated only single amount of tooth movement; the force systems produced by smaller or larger increments may differ.

## Conclusions

- 1. 3D-printed aligners from TC-85 generated significantly lower forces and moments than thermoformed aligners ATMOS® or Zendura FLX® aligners. The forces and moments were also more consistent and predictable with TC-85 versus ATMOS® or Zendura FLX®.
- 2. Attachments consistently aid in producing extrusive forces with thermoformed and 3D-printed aligners.
- 3. Extrusive forces can be produced in the absence of attachments when using pressure columns with 3D-printed aligners from TC-85.
- 4. Force systems created by movement of even a single tooth can be unpredictable. There were unplanned reciprocal forces and moments generated on the tooth planned for movement as well as on the adjacent tooth.

## Data Availability

All data generated or analysed during this study are included in this published article in the form of tables and figures.
